# Multiscale Mechanical Performance of Wood: From Nano- to Macro-Scale across Structure Hierarchy and Size Effects

**DOI:** 10.3390/nano12071139

**Published:** 2022-03-29

**Authors:** Yuri I. Golovin, Alexander A. Gusev, Dmitry Yu. Golovin, Sergey M. Matveev, Inna A. Vasyukova

**Affiliations:** 1 Institute “Nanotechnology and Nanomaterials”, G.R. Derzhavin Tambov State University, 392000 Tambov, Russia; yugolovin@yandex.ru (Y.I.G.); tarlin@yandex.ru (D.Y.G.); vasyukovaia@gmail.com (I.A.V.); 2 Department of Chemical Enzymology, School of Chemistry, Lomonosov Moscow State University, 119991 Moscow, Russia; 3 Research and Educational Center “Sustainable Development of the Forest Complex”, Voronezh State Forestry University Named after G.F. Morozov, 394087 Voronezh, Russia; lisovod@bk.ru; 4 Department of Functional Nanosystems and High-Temperature Materials, National University of Science and Technology “MISIS”, 119991 Moscow, Russia

**Keywords:** wood, nano-, micro-, meso-, and macro-structure, multiscale mechanical properties, size effects, Hall-Petch law, dendrochronology

## Abstract

This review describes methods and results of studying the mechanical properties of wood at all scales: from nano- to macro-scale. The connection between the mechanical properties of material and its structure at all these levels is explored. It is shown that the existing size effects in the mechanical properties of wood, in a range of the characteristic sizes of the structure of about six orders of magnitude, correspond to the empirical Hall-Petch relation. This “law” was revealed more than 60 years ago in metals and alloys and later in other materials. The nature, as well as the particular type of the size dependences in different classes of materials can vary, but the general trend, “the smaller the stronger”, remains true both for wood and for other cellulose-containing materials. The possible mechanisms of the size effects in wood are being discussed. The correlations between the mechanical and thermophysical properties of wood are described. Several examples are used to demonstrate the possibility to forecast the macromechanical properties of wood by means of contactless thermographic express methods based on measuring temperature diffusivity. The research technique for dendrochronological and dendroclimatological studies by means of the analysis of microhardness and Young’s modulus radial dependences in annual growth rings is described.

## 1. Introduction

**Figure 1:**
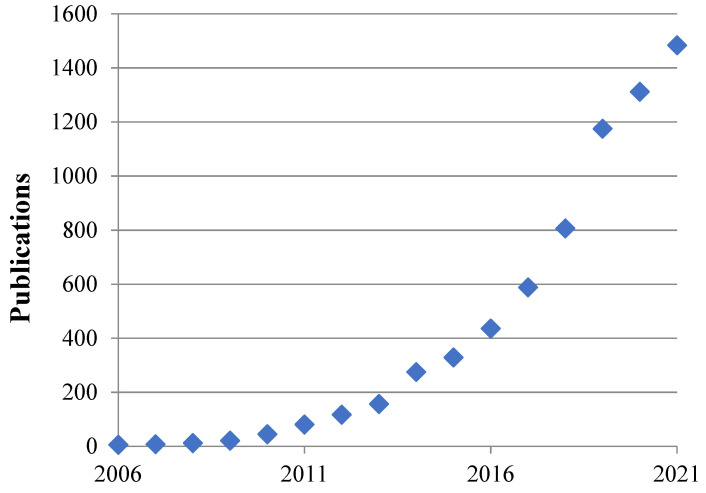
Growth dynamics of the number of research papers on the structure, properties, and applications of the materials comprising natural nano- and micro-fibers. Data obtained from Scopus using the following search parameters “TITLE-ABS-KEY” with keywords «Nanocellulose or micro-cellulose» on 28 February 2022.

Interest in wood and other cellulose-containing materials, composites in particular, had considerably increased by the beginning of the 21st century. The studies of wood nano- and micro-structures have especially intensified in the last decade (Figure 1) [1,2,3,4,5]. Several reasons can be named. Mineral resources (especially various metallic and nonmetallic materials, coal, oil, and natural gas) are being extracted at continually rising rates, and open-cycle processing technologies create ever-growing volumes of industrial and household waste. This poses a threat to the biosphere because of the environmental pollution and increased carbon dioxide concentration in the atmosphere, while only a fraction of the manufactured materials are recycled and reused. The situation is aggravated by a sharp increase in polymer material manufacturing for packaging, which are seldom recycled and mostly non-biodegradable. The surging pressure on the environment requires more and more efforts for its neutralization.

In this regard, a wider use of biogenic materials as well as substituting them for traditional ones seems a promising step. Such cellulose-containing substances as modified wood and various agricultural vegetable wastes, and especially the nano- and micro-cellulose they contain, offer the best potential for numerous applications.

Cellulose is the most common natural linear polymer polysaccharide (C_6_H_10_O_5_)*_n_* in the biosphere. The materials formed on its basis provide vast advantages:

1. Unlike the majority of extracted mineral resources, cellulose-containing materials have sustainable and renewable sources, namely forests, field crops, and aqua cultures [1,3,4,5,6,7];
2. These materials are multifunctional; they can be used in construction and industrial manufacturing [5,8], for producing cardboard, paper, packaging [9,10,11,12], and textile goods [13,14], in electronics [15], photonics [16], and energetics [17,18], in environmental remediation and wastewater treatment [5,19,20,21,22], medicine [23,24,25,26,27], military [28] and household applications, and in many other spheres [1,3,4,5,29];
3. Wood, cellulose-containing plant materials, and bio-composites are gaining more and more popularity each year. Among their most attractive features we should name their environmental friendliness, biodegradability, after-service “self-destruction” that leaves no toxic products [1,2,3,4,5], and their ability to be modified [30];
4. These materials are perfect for creating a closed carbon cycle, which does not increase the carbon dioxide content in the atmosphere [1,2,3,6], and it is a well known fact that this gas contributes to the greenhouse effect and to the average annual temperature growth;
5. Nano- and microstructural components in the wood structure (nanocrystals, nanofibrils, cellulose microfibers) possess mechanical properties (tensile strength *σ_b_*, Young’s modulus *E*, etc.) comparable with, and even exceeding the same properties of such high strength construction materials as steels, titanium, and aluminum-based alloys. Additionally, if we take into consideration their lower density *ρ* (~1.5 g/cm^3^ in nanocellulose vs. ~8 g/cm^3^ in steels, ~4.5 g/cm^3^ in titanium-based, and ~2.8 g/cm^3^ in aluminum-based alloys), then we discover that the specific values of strength *σ_b_/ρ* and stiffness *E/ρ* of nano-/microcellulose can exceed manifold those of steels and alloys;
6. Finally, they are manufacturable, non-toxic, and comparatively inexpensive.

Certainly, wood and other cellulose-containing materials have several disadvantages. They require special treatment, as they are flammable and hygroscopic. High humidity makes them lose some of their strength properties, while low humidity causes deformation. They succumb to rot and unwanted biodegradation. Besides, in their original, state the mechanical properties in every sort of wood strongly depend on the conditions of its growth, usage, and testing humidity, the structure of cell walls and annual growth rings, proportion of young and mature wood, stress condition, size of the sample or stressed area, and also the direction, rate, and duration of load application. The aforementioned considerations have obstructed identifying the universal patterns that form the mechanical properties of different species of wood. Nevertheless, some generalizations can be derived from the literature and from accumulated experience, as outlined below.

**Figure 2:**
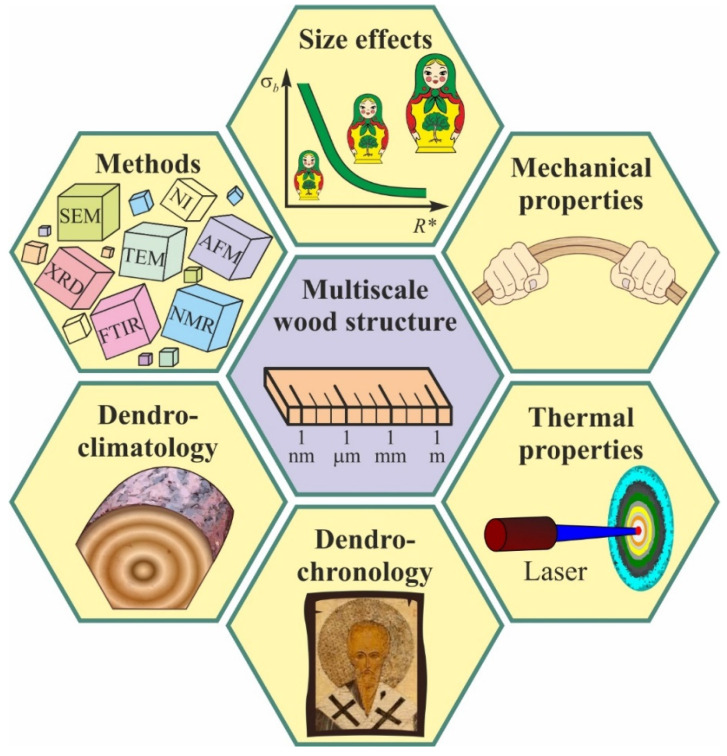
The main scopes of the review.

In the present review, we explore the methods and results of a multiscale study of the mechanical properties of various wood species, in connection with peculiarities of their nano-, micro-, and meso-scale structural levels of material organization. The analysis of literature data shows that, in a huge range of characteristic sizes of the structural units (about six orders of magnitude), mechanical properties of wood generally follow the Hall-Petch relation, which is well known in material science. The possibilities for non-destructive assessment of the mechanical properties of wood by means of contactless measurement of the temperature diffusivity tensor components are discussed, as well as using the scanning nanoindentation method for evaluating woods’ micromechanical characteristics, in order to obtain dendrochronological and dendroclimatological data. The main scopes of the review are presented in Figure 2.

## 2. The Hierarchical Structure of Wood

**Figure 3:**
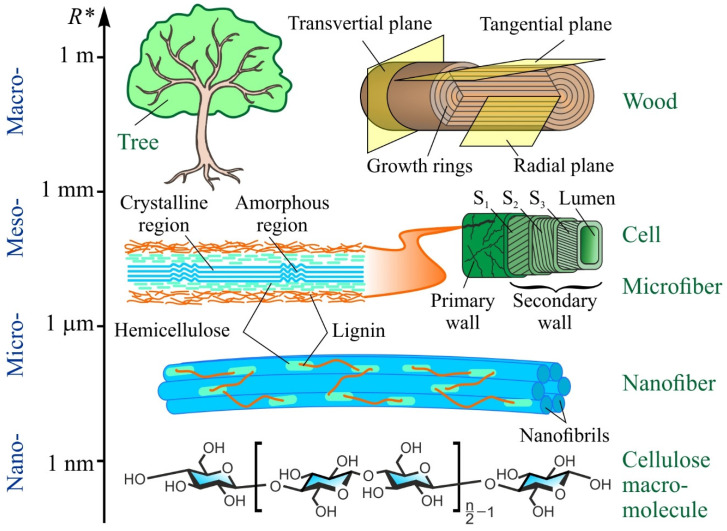
Scale hierarchy in wood structure and its main components. R* is the characteristic size.

From the point of view of material science, wood is a hierarchically organized natural composite with a complicated structure and a clear heterogeneity and anisotropy of all its properties, as well as an ability to regenerate [5,31,32,33,34,35]. In the wood architecture, one can distinguish, though only tenuously, several size and hierarchical levels (Figure 3), namely atomic–molecular, nano- (nanocrystals, nanofibrils), micro- (microfibers, cell walls), meso- (cells, large vessels, radial rays), and macro-level (annual growth rings, macroscopic structural defects, cracks, etc.) [31,32,33,35]. They all contribute to forming the complex of physical, chemical, and mechanical properties [31,36]. A large range of the characteristic size of the structural components of wood (about six to eight orders of magnitude) and a wide scope of tasks and questions emerging from the study of this material all require a varied arsenal of research techniques and means to implement them. They will be briefly analyzed in the following section.

Identification of patterns in the formation of the macro-properties of wood, as derived from its nano-, micro, meso-, and macrostructure, is the most important task in wood science. There are many reasons for the interest in the relations between macromechanical properties of wood and its nano- and microstructures, as well as physical characteristics, thermal characteristics in particular. Let us enumerate the most important ones. Firstly, the relevant patterns help to elucidate the nature and mechanisms of formation of the parameters most significant for practical applications of wood in the macroscale, i.e., its mechanical and thermal properties. Secondly, nanomechanical strength properties, being much higher than those at the micro- and macro-scale, indicate the potential for strengthening, which may approach the ultimate tensile strength of nanocrystalline cellulose (~10 GPa). Thirdly, the increased use of composite and nanocomposite materials in different spheres of engineering, construction, biochemical technologies, and medicine paves the way to replacing traditional metals and alloys with more lightweight and ecologically friendly composites. For example, the bodies of the most recent Boeing and Airbus airplane models consist, by weight, of more than 50% of fiber-reinforced composites. Their popularity in the auto industry, shipbuilding, sports equipment manufacturing, etc. is growing fast. However, glass and basalt fibers used for composite reinforcement, not to mention carbon micro- and nano-fibers, have some adverse properties from the ecological point of view; they are quite expensive and still unable to conquer the wide market for consumer goods. Cellulose fiber is by about an order of magnitude less expensive than fibrous glass while having almost the same mechanical characteristics. Therefore, it is important to understand the nature of strength and damage mechanisms in microcellulose fibers, and to find approaches to improve their strength, thus enhancing the properties of textiles, non-woven materials, and the composites they are used to reinforce. When correlations are revealed between the mechanical characteristics and other physical properties, for example thermal properties, this information will be of great use for developing non-destructive contactless thermophysical methods for evaluating mechanical characteristics, instead of applying labor-intensive destructive techniques. Fourthly and finally, many tree species have a lifespan of several hundred or even thousands of years, with sequoia as an example. In their nano-, micro-, meso-, and macro-structure they accumulate a vast amount of information about the climatic conditions during their growth and about ecological catastrophes they have witnessed. The variations in composition and structure are inevitably reflected in the local physico-mechanical properties of wood. This natural archive can serve as a valuable source of information for climatology and for dating various events in earth’s history (dendrochronology).

## 3. Methods of Studying the Structural and Mechanical Properties of Wood at Various Levels of Scale

The aim of the classic wood science is to discover and describe the dependence of woods’ macromechanical, physico-chemical, and service properties on its inner structural characteristics, humidity, and external thermodynamic factors [6,37,38]. Wood type classification and its grading, according to mechanical properties, is an important pragmatic task [39]. Since the end of the last century, more and more attention is being paid to the fine structure of wood at the nanoscale. This interest was brought forward, on the one hand, by the growth in nanotechnology and nanometrology, and on the other hand by realization of what untapped resources are hidden at the nanoscale.

In the recent 15–20 years, numerous modern methods and means traditionally used in solid state physics and material science are being applied for studying the micro-structure and physico-chemical properties of wood [2,6,33,35,40].

**Figure 4:**
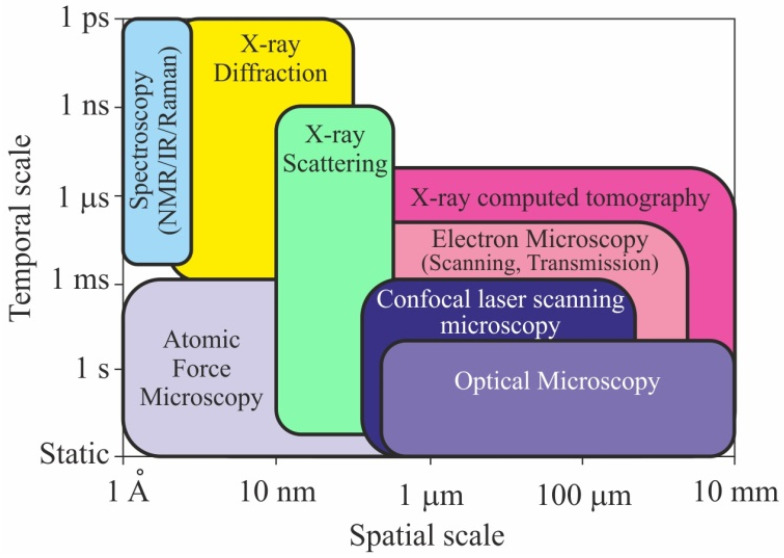
Schematic of the spatiotemporal ranges of the most popular physical methods for studying wood structures. Adapted with permission from Ref. [32]. Copyright 2021, Wiley-VCH.

Micro-structure is studied by means of transmission and scanning electron microscopy, scanning probes (mostly atomic force), confocal laser, and optical microscopy in various modes. Numerous X-ray methods are used to determine the composition and the parameters of atomic- and micro-structures. The character and degree of order of cellulose molecules in nano-fibers, the angle between the micro-fibers and the long axis of the cell, are determined by X-ray diffractometry and microtomography, as well as small-angle (SAXS) and wide-angle (WAXS) X-ray scattering. Elemental and molecular composition is revealed by spectroscopic methods, such as X-ray fluorescence, various types of spectroscopy such as infrared (IR), Fourier transform IR (FTIR), Raman, Brillouin, nuclear magnetic resonance (NMR), and other analytical methods. Together, they cover a huge spatiotemporal range of structures and events in them, namely more than twelve orders of magnitude in time and about eight orders of magnitude in length (Figure 4) [32]. The comparative analysis of the most widely employed physical methods for studying the molecular, sub-cellular, and cellular structures of wood can be found in most recent reviews [32,33,34,41].

To study mechanical properties at the nano- and micro-scale, a number of nano-/micro-mechanical testing (SSMT—small scale mechanical testing) methods [42,43,44,45] are employed. Atomic force microscopy (AFM) [46,47,48,49] and nano-indentometry (NI) [50,51,52,53,54,55,56,57,58,59] can be named as the most widely used ones.

**Figure 5:**
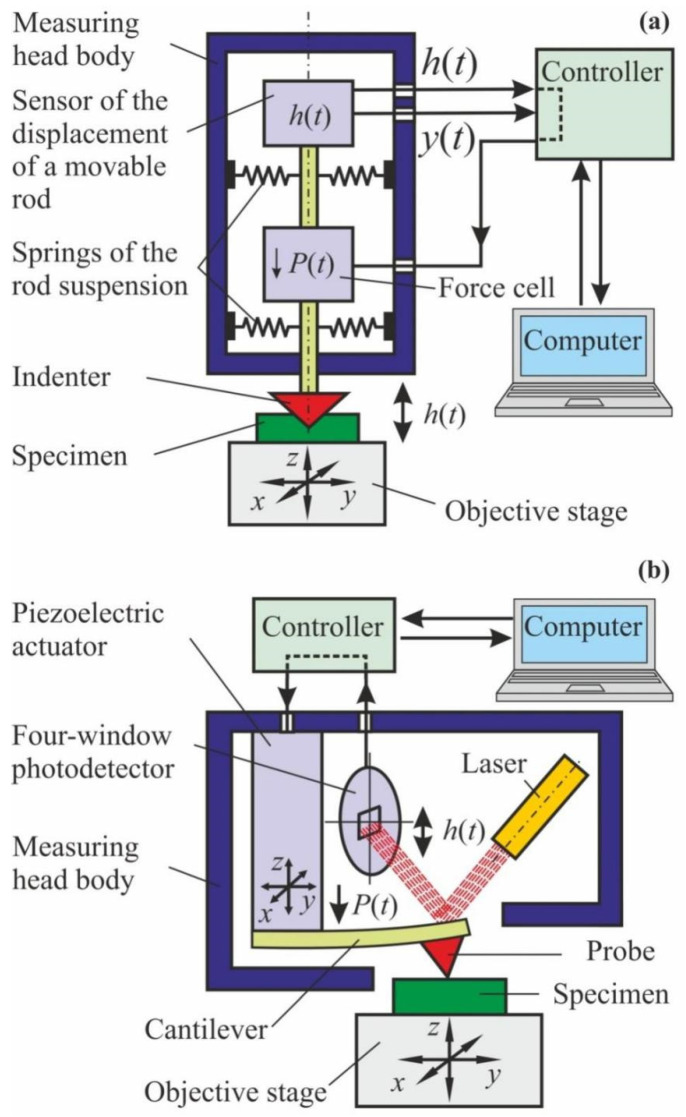
Schematic diagrams of (**a**) the nano-indentometer and (**b**) the atomic-force microscope.

**Figure 6:**
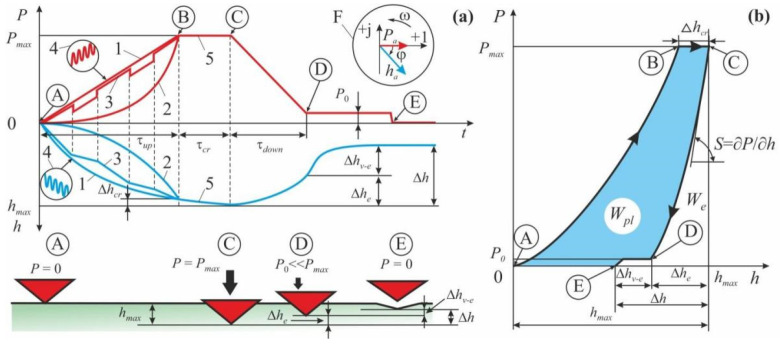
Two methods of representing the data obtained by means of normal nanoindentation: (**a**) as kinetic curves *P*(*t*) and *h*(*t*); (**b**) as a *P−h*-diagram (*P*—force, *h*—indenter displacement). Adapted with permission from Ref. [45]. Copyright 2021, Springer Nature. The circled letters (from A to E) mark the characteristic points on the loading curves and the indenter position relative to the sample surface. Inset F shows the vector diagram depicting correlation in the complex plane between the vectors of oscillating force and the resulting indenter displacement in the CSM method. Five loading regimes are marked from 1 to 5. The indices at *P* and *h* mean as follows: *up*—increase; *cr*—creep; *down*—drop; *e*—elasticity; *v−e*—viscoelasticity; *max*—maximum value; *W_e_*—elastic energy; *W_pl_*—energy absorbed and dissipated by the sample in a single load–unload cycle.

They have similar structure flowcharts (see Figure 5) and capabilities [45,50,52]. In both cases, a high precision driver brings a probe, with the radius of its curve being from a few (in AFM) to a few tens (in NI) of nanometers, close to the studied surface and the probe starts interacting with it. The force *P* and penetration depth *h* of the probe are measured continuously, and their alteration kinetics are registered throughout the testing cycle (Figure 6a). Most commonly a *P*–*h* diagram (similar to a σ–ε diagram created during macro-testing) is built using the obtained data (Figure 6b), and standardized algorithms are applied to calculate about ten various mechanical characteristics, such as Young’s modulus, contact stiffness, hardness, fracture toughness, creep rate, etc., at the nano-/micro-scale. In NI, a three-sided diamond Berkovich tip is used, as it is better calibrated from the point of view of the real tip geometry than the one used in AFM, thus providing more accurate and reliable quantitative data. Among the variety of proposed techniques for mechanical characteristics extraction from raw data, the method proposed and developed by Oliver and Pharr [60,61,62] has become the most widely used and has been incorporated into ISO standard [63], so that this method has been used for processing all NI experiments described in this review.

SSMT methods were used to examine the mechanical properties of individual cellulose nanofibrils and microfibers [64,65,66,67,68], cell walls [32,69,70,71,72,73,74,75], layers of early and late wood in annual growth rings [74], and to obtain plenty of other interesting data. However, there are very few papers that compare and analyze several scale levels at once [76,77,78,79]. Thus, connection between the properties of individual nano- and micro-structural elements of wood and their influence upon macro-mechanical characteristics cannot yet be traced.

The analysis of the structure and role of annual growth rings in shaping wood macro-properties requires, at the very least, one-dimensional, or better yet, two-dimensional scanning of certain physical characteristics. Three-dimensional imaging can be applied as well. Dating archaeological finds, works of art, climate changes and events, and ecological catastrophes based on the changes in growth ring structure and width is a separate issue. These approaches are known as dendrochronology, dendroecology, and dendroclimatology, respectively. The width of rings, proportion of early wood (EW) to late wood (LW), and changes in their morphology reflect the specific growing conditions each long-lived plant witnessed during each vegetation season.

While microstructure and physico-chemical properties of wood are studied with elaborate modern equipment, examination and analysis of annual growth rings for dendrochronological and dendroclimatological applications is carried out using simple optical methods, where primary information is derived from the difference in reflectivity between EW and LW. Quite often, the same approach is employed while assessing wood strength and other service properties. A detailed description of dendrochronological methods developed by the beginning of this century is given in [80]. These methods reveal only geometrical and morphological characteristics of the studied object (annual growth rings width, proportion of EW and LW in them, variations from ring to ring, etc.) and allow for comparison between the data obtained by different methods.

Numerous attempts have been made to improve traditional dendrochronological methods, mostly by modifications introduced to the sample preparation techniques, staining, use of blue light instead of white, application of computer vision technologies, and mathematical data processing (see e.g., [81,82,83,84,85,86,87,88]). However, despite this, the capabilities of the approach based on the analysis of transversal section images and photodensitometry remain severely limited, as the reflective optical properties of wood are variable and their connection with other wood characteristics, such as mechanical and thermal, are either ambiguous or very weak.

In order to expand the capabilities of the analysis of mechanical properties in their connection with the architecture of wood ring structures, the following methods have been used: two-dimensional mapping of properties on cross-sections of tree trunks by AFM methods [89,90,91,92] and NI [93] scanning, 3D X-ray [94,95] and NMR tomography [96], and synchrotron-based X-ray microscopy [97]. However, these methods are complicated, labor-intensive and require expensive or unique equipment; therefore, they are used only sporadically. The method of X-ray densitometry [98] presents fewer difficulties, but it requires access from both sides to a perfectly flat sample cut exactly perpendicular to the long axis of wood cells.

It should be noted that the mechanical properties of wood and cellulose-containing materials show a significant dependence on the rate of monotonous loading, oscillating load frequency, and duration of load application. They can vary between samples and change over time in a significantly greater range than in similarly structured technogenic composites (e.g., in glass and carbon fiber-reinforced plastics) [2,5,40,41]. Such variability of properties makes identification of common regularities in their formation even more challenging.

To sum up, we should mention that, thus far, the overall links between the properties of all the scales and hierarchical levels—from cellulose nanocrystals (CN) to macro-samples—require additional study. However, there is a considerable volume of information on every individual level. The following sections present the examples of the most representative data from the lowest to the highest scale levels of the structure.

## 4. Nanocellulose and Elementary Nanofibrils

Cellulose is the most common natural polymer and the major structural component that provides strength to wood and stems of grass, reed, bamboo, etc. [5,40]. Cellulose is a macro-molecular polysaccharide (C_6_H_10_O_5_)*_n_*, consisting of linear chains of several tens to many hundred *n* of β-(1→4) linked glucose molecules (Figure 2). In its origin, cellulose can belong to three types: plant, regenerated, and bacterial [5]. The current state of affairs in extraction and functionalization of cellulose nanofibers is described in Handbook [5] and in the most recent reviews [99,100,101,102].

Cellulose molecules easily form nanocrystals with a lateral size of 3–10 nm and being 100–300 nm long. These nanocrystals form nanofibrils 5–20 nm in diameter and up to many hundreds of nanometers long. Inside, nanofibril cellulose is present in an amorphous–crystalline state as a series of alternating domains. The amorphous phase, to some extent, reduces the strength of the nanofibril, but makes it more supple and elastic. The most typical structural characteristics of nanocellulose-based formations are presented in Table 1 [67].

Mechanical properties of nanocrystalline cellulose have been characterized by various methods, including calculations of bond strength inside macro-molecules and between them, computer generated simulations, processing data from IR and Raman spectroscopy, AFM, WAXS, and others [5,40,65,68]. A brief overview of the mechanical characteristics of nanocellulose is given in Table 2 [67]. The variability of data is explained by specific characteristics of the calculation schemes, models, raw data processing algorithms, and also by the difficulty of carrying out direct measurements at the nanoscale. The differences in age, structure, and origin of wood affect the experimental results as well. Besides, the mechanical properties of nanocellulose samples depend on their size significantly. For instance, transversal Young modulus reduction by a factor of 1.6 has been reported in [78] for increasing NC diameter from 2.5 nm to 6.5 nm.

## 5. Cellulose Microfibers

The typical hierarchy of wood structure at higher levels continues with nano- and micro-fibers. They are formed by elementary nanofibrils, mainly due to hydrogen bonds. Nanofibrils form strands surrounded by a matrix composed of lignin (an aromatic polymer polyphenol), hemicellulose (low molecular weight branched polysaccharide), pectin (gel-forming polysaccharide), and water [5,40,68]. Cellulose content in the fibers can vary in a wide range. For example, it is 40–60% in the wood fibers of various species and can exceed 96% in cotton fibers [5,23,24,31,68].

**Figure 7:**
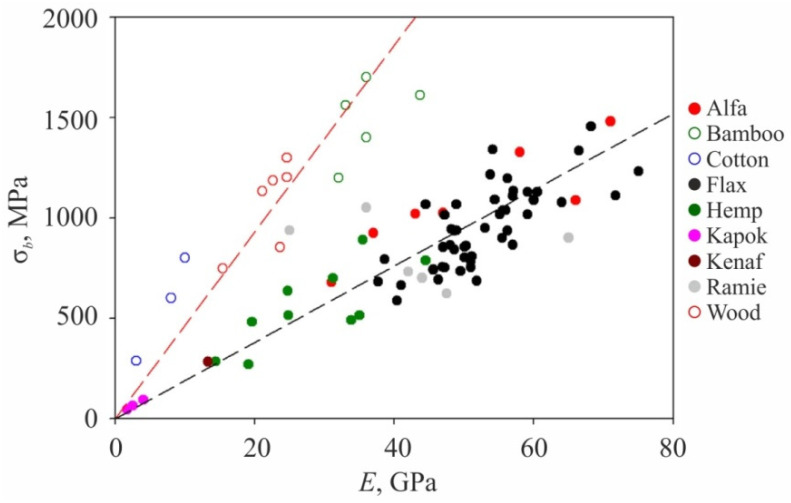
Dependence of cellulose fiber tensile strength upon Young modulus for various plant materials. Adapted with permission from Ref. [104]. Copyright 2018, Elsevier.

Nano- and micro-structures of cellulose materials and their properties strongly depend upon the specifics of interaction between nanocrystals in elementary fibrils and the ordering and binding of the latter in nano- and micro-fibers [103,104,105,106,107]. Mechanical, strength in particular, properties of cellulose nano- and micro-structures are structure sensitive, just as those of most other other organic and non-organic materials. In turn, their morphology and inner structure depend upon plant species, their growth conditions, and cellulose extraction technology. The dominant role in determining fiber properties belongs to the cellulose content in nanofiber, the degree of its crystallinity, and specifics of nanofiber binding at the material. The angle between the nanofibril axis and nanofiber or cell axis has significant impact too. A comprehensive review [104] contains various data concerning morphology, microstructure, and mechanical properties of micro-fibers of various origin (Figure 7) and examples of their application for polymer composite reinforcement.

The strongest of the studied micro-cellulose fibrils have demonstrated Young modulus *E* = 75–85 GPa and tensile strength σ*_b_* = 1.6–1.7 GPa, so that the ratio *E*/σ*_b_* ≈ 50. One of the possible techniques allowing researchers to reach such high mechanical properties is described in [66]. The authors have used the efficient technique of double hydrodynamic ordering of nanocrystals and nanofibrils to produce the fibers with diameter 6–8 μm. Their tensile strength reached 1.1 GPa. Nanofibril cross-linking has increased the fibers’ strength up to σ*_b_* = 1.57 GPa.

As follows from fundamental considerations, the theoretical strength of any defect-free material can reach 0.1 *E*, while the strongest micro-cellulose fibers mentioned above have values around 0.015–0.020 *E*. Hence, even the strongest studied micro-fibers have the potential of increasing their strength by 3–5 times.

It should be mentioned that the data concerning cellulose micro-fibers mechanical properties differ significantly depending on the measurement technique (see Table 3) [104]. Results obtained using AFM and NI are in agreement with each other regarding the measurement accuracy, despite using different probes and measurement techniques, so that they are just as reliable as the ones obtained using the undebatable method of uniaxial tension. Usually, its tensile strength is two to three times higher than compression strength or hardness [5,40,104,106,107], unlike void-free materials, where the reversed value is quite typical. For example, the Tabor ratio for metals is well known, where hardness is three times higher than the yield stress. We suppose that this difference is due to specific nanofibril behavior when subjected to tensile and compressive stress or in hardness measurements. In the first case, the molecular chains are strained and partially oriented along the fiber axis, which increases their strength. Indentation, on the other hand, used both in NI and AFM, promotes the arising of compression stress that causes micro-fibril bending, and micro-fibril buckling failure, which occurs earlier than their failure in uniaxial tension.

**Figure 8:**
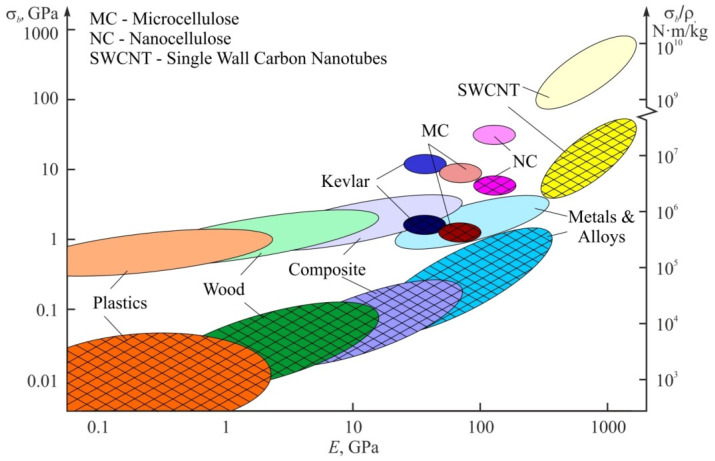
Mechanical properties of nanocellulose (NC) and cellulose microfibers (MC) in comparison to common and perspective constructional materials. Crosshatched areas denote absolute values of tensile strength *σ_b_*, while non-crosshatched ones are the strength normalized over material density *ρ*.

For many applications such as aviation, space aeronautics, the automotive industry, sport equipment etc., the most important mechanical characteristics are not absolute but specific ones, i.e., normalized on material density *ρ*. Figure 8 shows the absolute σ*_b_*, specific strength σ*_b_/ρ*, and Young modulus *E* for highly oriented cellulose nano- and micro-fibers when compared to those for macroscopic wood and other materials. As it can be seen, the specific strength of defect-free nanocellulose can be manifold higher than that of aluminum or titanium alloys or constructional steels. However, nano- and micro-cellulose materials are inferior to metals at thermal and crack resistance, failure deformation, and other related energy characteristics. Only some polymers such as kevlar and carbon microfibers can contest nanocellulose at specific strengths. Single-wall carbon nanotubes and graphene are manifold superior at specific strength to any other known material.

Lastly, let us mention one more advantage of natural cellulose fibers produced from the wood. It is several times cheaper than the flax fibers and an order of magnitude cheaper than ecologically unsafe glass fibers widely used in composite reinforcement applications [104]. Highly ordered cellulose microfibers have nearly the same strength as glass fibers already and have a good prospects of further strength increases.

## 6. Cells and Cell Walls

While a tree grows, cellulose microfibers integrating with other components such as lignin, hemicellulose, pectin, water, etc., form walls of cells that are highly elongated in the direction of tree trunk axis. Several layers are discerned within cell wall including primary wall and multilayered secondary walls that usually consists of three layers, named S1, S2, and S3, which differ in the angle μ between cellulose microfibers and cell long axis. The secondary wall provides the main contribution to cell stiffness and mechanical strength. Cell size diminishes, cell wall width increases, and the cross-section of internal capillary reduces while going from early wood (EW) that is a part of the annual ring formed at the first stage of vegetation, to late wood (LW), formed at the second stage.

**Figure 9:**
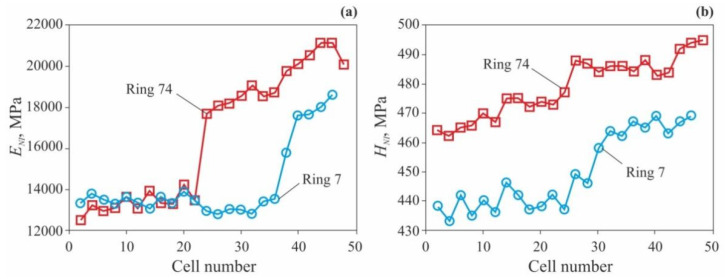
Dependencies of cell wall longitude Young modulus *E_NI_* (**a**) and nanohardness *H_NI_* (**b**) upon cell sequential number in the annual growth ring for two rings with ages of 7 and 74 years. Adapted with permission from Ref. [74]. Copyright 2020, PAN.

To study mechanical properties of wood cells, various SSMT methods are used [45], and the most widespread ones are AFM [89,92] and NI [70,71,74,75,93]. Let us present some typical examples of NI application with load *P_max_* = 0.1–1 mN to this problem. The authors of [74] studied radial dependence of cell wall longitude Young modulus *E_NI_* and nanohardness *H_NI_* in two annual rings of common pine (*Pinus sylvestris* L.) wood, corresponding to the ages of 7 and 74 years. As could be seen at Figure 9a, in going from EW to LW, *E_NI_* increases by nearly 50%, while *H_NI_* (Figure 9b) increases by just 5–7%.

A number of other papers report similar data supporting that cell wall nanohardness varies not too much at different layers, rings, or even tree species. For instance, the following results are reported: *H_NI_* = 0.35–0.42 GPa for *Pinus massoniana* Lamb. In [107], *H_NI_* = 0.41–0.53 GPa for *Masson pine*, coinciding within the measurement accuracy for EW and LW in [108] and *H_NI_* = 0.34–0.54 GPa for *Pinus taeda*, not discerning EW and LW, in [109]. Similar results are reported for *H_NI_* in cell walls junction through the middle lamella for *Norway spruce*. Nanohardness in the cell corner middle lamella was estimated to be 0.34 ± 0.16 GPa [110].

The NI technique allows more detailed measuring cell wall elastic properties and determining the main components of elasticity tensor. So, the measured value of Young modulus of secondary wall S2 has been reported to be 26.3 GPa in the longitudinal direction and 4.5 GPa in the lateral one [111].

The most informative experiments are carried out in situ in an electron microscope column using a sharp indenter or flat piston [112]. Simultaneous recording of loading diagrams and obtaining visual information concerning the nano-/micro-structure evolution allow the studying of the micro-mechanisms of deformation and failure [113].

There are a number of papers studying the mechanical properties of micro-pillars cut from cell walls by focused ion beams (FIB). The pillars were subjected to uniaxial compression in a scanning electron microscope column (SEM) [114,115]. It allows simultaneous recording of σ-ε diagram and specific features of micro-pillar deformation.

Such works are not numerous due to high labor content and rather complex and expensive equipment required for sample preparation. However, they provide direct confirmation of quantitative information obtained using NI, allow for obtaining unique information concerning various deformation modes, buckling, and failure mechanisms of cell walls, and verification of various behavior models of wood hierarchic structures under load.

## 7. Annual Growth Rings

As follows from the data presented in the section above, cell wall Young modulus *E_NI_* does not vary significantly with the indent location, be it late or early wood layer, secondary wall, or cell conjugation region with middle lamella. The ring age, weather conditions during its formation, or particular tree species does not change it more than by a factor of 1.5–2. Nanohardness *H_NI_* dependence upon these factors is even weaker. Nevertheless, macromechanical properties woods of differing origin can differ manifold reaching up to an order of magnitude or even more. Evidently, this weak correlation between nano- and macro-properties is due to the difference in cell wall thickness, the relative share of late wood, and the number of large tracheides and other wood structure elements which reduce wood macroscopic strength. To close the gap between nano- and macro- scale mechanical properties, the nanoindentation tests were carried out at loads ranging from 5 to 500 mN and reported in the set of papers [116,117,118,119], unlike the 0.1–1 mN range usually used in studying cell walls. It extended the deformed region over the whole cell or several cells up to 50–150 μm, as opposed to precise targeting at the cell wall.

The values of Young modulus *E_eff_* and microhardness *H_eff_* obtained this way can be considered as effective, as long as, being the result of averaging over the cells structure, they incorporate not only mechanical properties of cell walls but also their thickness, material porosity, and microdefects, just as in macroscopic mechanical tests. However, indentation size was at least an order of magnitude less than annual ring width. It allowed obtaining *E_eff_* and *H_eff_* spatial distributions across several annual rings.

**Figure 10:**
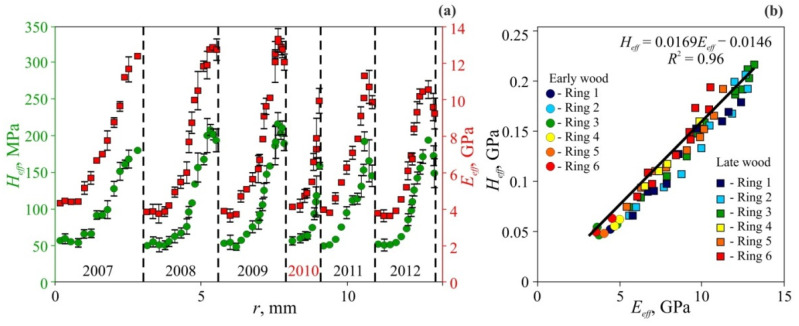
Micromechanical properties of common pine annual growth rings measured at *P_max_* = 500 mN [116]. (**a**) Spatial dependencies of *H_eff_* and *E_eff_* over radial distance *r* for six successive rings. Ring boundaries are shown using dashed lines. The extraordinarily draughty 2010 year is highlighted by red color. (**b**) Dependence of hardness *H_eff_* upon Young modulus *E_eff_* for six successive rings.

**Figure 11:**
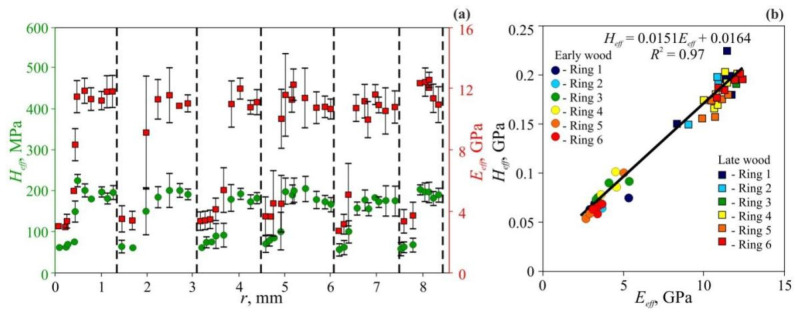
Micromechanical properties of pedunculate oak annual growth rings measured at *P_max_* = 500 mN [116]. (**a**) Spatial dependencies of *H_eff_* and *E_eff_* over radial distance *r* for six successive rings. Ring boundaries are shown using dashed lines. (**b**) Dependence of hardness *H_eff_* upon Young modulus *E_eff_* for six successive rings.

Typical *E_eff_* and *H_eff_* radial dependencies measured at cross-sections of common pine (*Pinus sylvestris* L.), which represent coniferous trees, are shown at Figure 10, and pedunculate oak (*Quercus robur* L.), which represent hardwood trees, are shown at Figure 11 [116,117]. As could be seen, both species manifest pronounced periodicity of local mechanical properties. Positions of abrupt changes in *E_eff_* and *H_eff_* coincide with annual growth ring boundaries determined by wood color change using the standard optical method. Changes of *E_eff_* and *H_eff_* in transition from EW to LW within each annual ring are gradual in pine and abrupt in oak. The linear dependence between *E_eff_* and *H_eff_* with almost the same slope *m* = 0.017 ± 0.002 has been observed (see Figure 10b and Figure 11b). In other words, the ductility ratio DR = *E_eff_*/*H_eff_* is found to be around 60 for both tree species. This value is quite typical for many other species, for example DR in gum-tree lies within the 54 to 68 range, with an average of 61 [120], and in beech it is around 55 [121].

As could be seen at Figure 10a and Figure 11a, *E_eff_* and *H_eff_* vary not too much within each EW layer and across several EW layers (relative variance of both values is around 10–15%), despite that the weather conditions during layer growth may differ significantly. For instance, the year 2010 was very dry. It affected the ring width *w_o_*, so that it is less than half of an average one, however, *E_eff_* and *H_eff_* values for EW are almost the same as appropriate values for other years (Figure 10a). The lateral size of EW cells in different rings does not differ significantly either, but cell wall width does. Thus, ring width variation is mainly due to the difference in cell morphology and its quantity in the layer, while the mechanical properties of cell wall material are almost the same.

As follows from the data presented, the ratio of averaged Young moduli for LW and EW is around 3.1 for pine and 3.5 for oak. The ratio of averaged hardness for LW and EW is close to those measurements, and equal 3.7 for pine and 3.0 for oak. These values stay for average values calculated for individual rings, but in some years, they can differ from the mean substantially. Obviously, such variations are due to anomalous weather conditions during these years, and narrower growth rings corresponding to these years evidence the same. However, year-to-year variation in mechanical properties is much higher than in annual ring widths, which is usually used for climate reconstruction. Thus, *E_eff_* and *H_eff_* measurements can be a much more sensitive and accurate dendroclimatological method than annual growth rings width measurement.

The values of *E_eff_* and *H_eff_* measured as described above are two to three times lower than those of cell walls. This is expected as long as the former are affected by material porosity. However, *H_eff_* is two to three times higher than macroscopic Brinell hardness [8,76]. It can be formally qualified as size effect (SE) in wood mechanical properties. However, determining the relative contributions of void-free material properties, porosity, and the related differences in deformation mechanisms in such SEs requires separate research.

## 8. Mechanical Properties of Wood at Macroscopic Scale

The largest part of the literature discussing mechanical properties of the wood concerns properties at the macroscopic scale [4,8,32,35,122]. As long as mechanical properties of the wood demonstrate prominent anisotropy, reference data typically comprise Young modulus, hardness, strength, and other properties for directions along and across fibers separately [35]. The methodological review of generally used approaches and experimental techniques of mechanical testing of the wood can be found in [123]. Standard methods of timber mechanical testing are described in [124]. Methods of timber strength classification can be found in [35,125].

The most general relationships between the mechanical properties and structure of the wood are discussed below. Going from juvenile wood (JW) that is formed during the first 5–20 years of tree growth to mature wood (MW), specific gravity, cell length and wall thickness, percentage of late wood, and strength increase, while fibril angle μ, moisture content *W*, and annual ring width *w* decrease. Moisture content *W* increases by 1% in the range between 10–12% and 50–60% results in a decrease in uniaxial compression strength by 5% and in uniaxial tensile strength by 2–2.5% both along and across fibers [126]. Young modulus across the fibers diminishes with growing *W* too but at the slower rate of around 1.5% per 1% of *W*. Typical tensile strength of softwoods (fir, pine, spruce, cedar, etc.) along the fibers is between 45 and 112 GPa, while that of hardwoods (beech, oak, maple, elm, etc.) is between 70 and 120 GPa [8]. Cooling from room temperature to –195 °C results in increase in compressive strength of dry wood with *W* = 12% by a factor of 2–2.5, while heating to 50 °C rises it by 10–20% [8]. Holding the wood under load for a long time diminishes its strength. So, it drops by 10–15% for an hour, by 20–25% for a month, and 30–35% for a year when compared to 1 min load [8]. Fracture toughness of macroscopic wood samples range from 250 kPa m^1/2^ for Western white pine to 517 kPa m^1/2^ for Yellow poplar [35]. In addition to the species, growth conditions and moisture content, structure defects significantly affect wood macromechanical properties [8,122,123]. Their variation can reach 15–35% from sample to sample due to such sensitivity. More detailed data concerning mechanical properties for various sample size and testing conditions can be found in [4,8,32,35,122,125,126] and Table 4 in Section 10.

## 9. Modification and Hardening of Wood and Cellulose

Drastic reduction of wood mechanical characteristics at increasing characteristic size of the samples stimulates development of various modification techniques for both macroscopic wood products [30,127,128] and nano- and micro-cellulose [5,129,130]. Several classes of wood modification techniques could be distinguished, including (a) chemical processing (acetylation, furfurylation, resin impregnation etc.); (b) thermally-based processing; (c) thermo–hydro–mechanical processing (surface densification); (d) microwaves, plasma, and laser light treatment; (e) mineralization; (f) biological treatment [30]. Besides improvement of mechanical properties, wood modification can be aimed at reducing water absorption or susceptibility to rotting and biodegradation, enhancing fire resistance or antiseptic properties, improving dimensional stability or resistance to acids or bases, ultraviolet radiation etc. Getting back to mechanical properties, let us mention some examples of wood modification leading to significant improvement of such properties. So, the widely used Compreg technique, consisting of wood compression before the resin is cured within the material, leads to considerable compressive strength increases that are even higher than wood density increases; tensile strength, and flexural strength increase less than its density increases [30]. As long as wood density can be increased up to a factor of 2–2.5 during this processing, its strength can be increased nearly twofold. At the same time, material toughness drops by 25–50%. The hardness can be raised by Compreg more substantially, and the factor of 10 to 20 has been reported [30]. Another modification technique is described in review [31]. The two-step process involves the partial removal of lignin and hemicellulose from the natural wood via a boiling process in an aqueous mixture of NaOH and Na_2_SO_3_, followed by hot-pressing, leading to the total collapse of cell walls and the complete densification of the natural wood with highly aligned cellulose nanofibers. The processed wood has a specific strength higher than that of most structural metals and alloys, making it low-cost and high-performance. More detailed information concerning techniques and results of wood modifications aimed at changing the mechanical and other service properties of the wood can be found in books [8,30,127], reviews [128], and original papers [131,132,133,134,135,136].

## 10. Size Effects in Wood

All the size effects in materials are usually divided into internal ones that depend on nano- and micro-structures of the object, and the external ones, depending on the size and shape of the sample, loading method, and size of the area under load. There is not much systematized information available regarding both types of SEs at different scale hierarchical levels of wood structure. In our survey, we present the most interesting and typical data. They are summed up in Table 4.

**Table 4:** Some mechanical properties of wood structure components.

Specimen		Young’s Modulus, GPa		Tensile Strength, GPa		Stress Strength, GPa		Hardness, GPa		Reference
	R*	‖	⊥	‖	⊥	‖	⊥	‖	⊥	
CNC (Cellulose Nanocrystals)	5–30 nm	140–160	15–30	8–10	~1					[67]
	3–20 nm	105–168		7.5–9						[103]
	5–70 nm	150–175								[129]
	~10 nm	110–220	10–50	7.5–7.7						[40]
CNF (Cellulose Nanofibril)	10–40 nm	30–40	10–15	0.8–1	~0.1					[67]
CMF (Cellulose Microfibers)	10–70 μm	120–140		0.75–1.08						[36]
	10–50 μm	12–27		0.3–1.4						[40]
	10–30 μm	15–27		0.55–1.3						[104]
	~10 μm	86		1.57						[66]
Cell wall										
*Pinus sylvestris* L. *Pinus massoniana* *Masson pine* *Pinus taeda*		17 ± 5						0.46 ± 0.03		[74]
								0.38 ± 0.04		[107]
								0.47 ± 0.06		[108]
								0.44 ± 0.1		[109]
EW layers										
*Pinus sylvestris* L.	~1 mm	4 ± 1						0.05 ± 0.01		[116]
*Quercus robur* L.		4 ± 1						0.08 ± 0.02		[116]
LW layers										
*Pinus sylvestris* L.	~1 mm	11 ± 2						0.18 ± 0.04		[116]
*Quercus robur* L.		12 ± 1						20 ± 0.02		[116]
Bulk wood										
Pine (misc.)		8.5–13.7		0.08–0.12	0.002–0.003	0.04–0.06	0.003–0.007			[8]
*Pinus sylvestris* L.								0.03–0.04	0.01–0.02	[116]
*Pinus sylvestris* L.								0.04–0.05		[117]
Oak (misc.)	10–1000 mm	10.3–13.9		0.08–0.16	0.003–0.007	0.04–0.06	0.006–0.009			[8]
*Quercus robur* L.								0.06–0.07		[117]

Legend: R*—characteristic size ‖—along the fibers ⊥—across the fibers.

As follows from Table 4, the strength of cellulose nanocrystals, assessed both by calculations and by experimental techniques, is 4.9–10 GPa, while the strength of nanofibrils with a diameter 3–15 nm is close to the lower end of this range [5,40,65,66,67,68]. These values exceed the strength of cellulose microfibers 8–12 µm in diameter, which is 0.5–1.65 GPa, by about one order of magnitude [5,40,66,104,106,107]. The typical nanohardness values *H_NI_* for cell walls with a thickness of 2–5 µm are about 0.3–0.5 GPa [70,71,72,73,74,75,107,108,109,110], which is 2–3 times less than the strength of cellulose microfibers. As it is shown in Figure 10a and Figure 11a, the effective values of microhardness, *H_eff_*, which take into account the porosity in layers EW and LW, are several times lower (from two to four times) than *H_NI_*. However, the *H_eff_* value is several times higher than Brinell macrohardness HB [76], and bending and uniaxial tensile strength obtained in macrotests [8].

It is evident that the values of effective Young’s modulus and hardness at meso- and macro-levels fall so dramatically not only because of the internal reasons defined by molecular and supramolecular structures, but also due to a great influence of nano-/micro-porosity of the material that should be attributed to the number of pores, capillaries, and larger tracheides with a high aspect ratio, ubiquitous in in the wood structure. Their presence results in several considerable differences between the mechanical behavior of wood and non-porous bodies. Firstly, Tabor’s rule, according to which the hardness of soft materials exceeds their yield stress or strength by about three times, is almost never met. On the contrary, in most wood species, their macroscopic hardness is several times lower than the yield stress and ultimate tensile strength determined for macroscopic samples. Apparently, in all similar events, the reason is that during indentation and uniaxial compression, the tested wood cell structure loses its stability long before the manifestation of plastic deformation and tensile rupture. These events strongly depend on the direction of the load application in relation to the long axis of the cell due to anisotropy of the mechanical properties of wood. In the longitudinal direction, they are higher by about an order of magnitude than in the transverse direction. It is difficult to provide an explanation for the various possible modes and mechanisms of wood deformation at the nano- and micro- scale because of insufficient experimental data. However, the quantitative information quoted above leads to the conclusion that there exist highly pronounced SEs in wood which cause a sharp decrease in strength/hardness from ~10 GPa in nanocrystalline cellulose to ~0.1 GPa or less in macrovolumes of wood. This means that all cellulose-containing materials have a large strengthening potential that can be realized through optimally organized nano- and micro-structures, and employment of relevant technologies.

**Figure 12:**
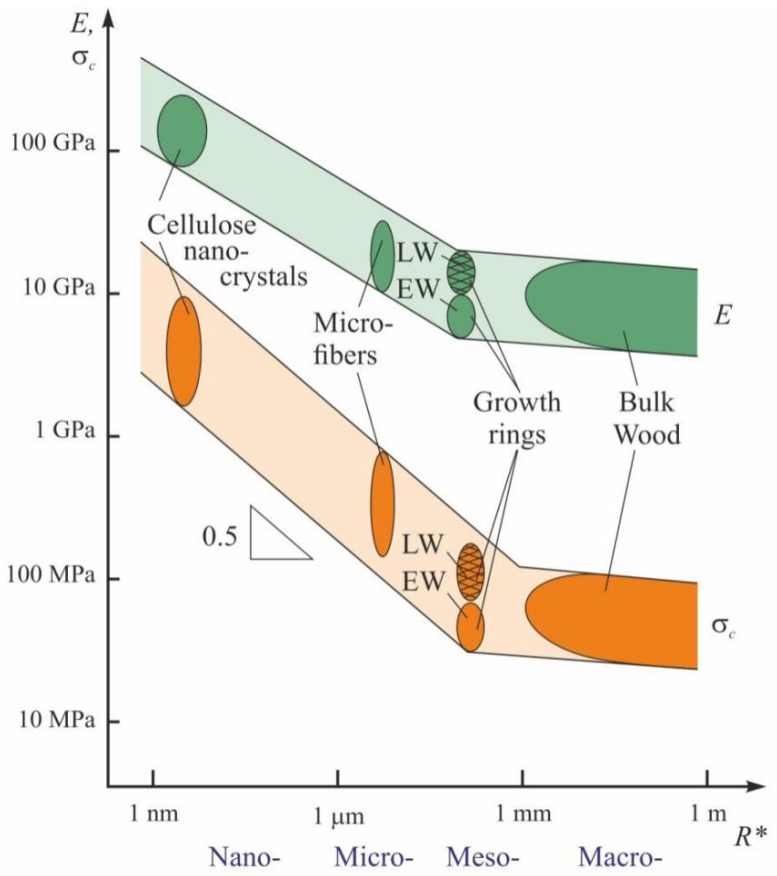
The size dependence of cellulose-containing materials strength. (Data collected by the authors). R* is the characteristic size.

Representation of the strength characteristics as a function of characteristic dimensions of the structure R* in double-logarithmic coordinates provides a distinctive hockey stick-shaped diagram (Figure 12). The descending part of the curve in the nano- and micro-domain has a slope close to−0.5, a feature it shares with Hall-Petch relation, which is well-known in materials science and described for the first time for polycrystalline metals more than 60 years ago [137,138,139]:

(1) $$\sigma_{y}=\sigma_{0}+A{\left(d\right)}^{-0.5}$$

where *σ_y_* is the yield stress, *d* is the crystallite size, *σ*_0_ and *A* are material constants. In most cases, the value of *A* that used to be called the Hall-Petch constant turned out to be deformation-dependent [139]. Later it was clarified that external dimensions affect the mechanical properties in the similar way, though the index of power may differ from 0.5 [45].

Relations, similar to (1) were discovered for hardness [140]

(2) $$H=H_{0}+A{\left(R^{*}\right)}^{-0.5}$$

where R* is the characteristic dimension of the locally deformed area, which, in the process of indentation with a Berkovich tip, is usually taken equal to the indentation depth *h_max_*.

The specific internal and external SEs have been studied not only in void-free poreless materials (metals, alloys, rocks, composites, etc.), but also in such porous materials as ceramics, solidified foams [141,142,143,144], and organic gels [145].

Due to a discrete character of damage accumulation in porous materials under load, the loading diagrams obtained in NI and in microsample deformation contain deformation jumps. They carry information about elementary events, their rate and statistics as a function of size of the area under load, deformation rate and other experiment conditions [143].

Obviously, the causes for drop in strength/hardness with increase in R* may differ in different groups of materials. Nevertheless, some similarities can be observed, for example, the principle “the smaller the stronger” works in wood as well, though the index of power for R* may vary within quite a wide range, namely from 0.2 to 1. Therefore, many authors suggest other dependences to account for SEs, such as 1/R*, ln(R*)/R* and others [146,147].

The softening mechanisms turning on with the increase in both internal and external characteristic size of the system require additional study of interrelations between the multilevel nano-/micro-structure of wood and its physico-mechanical properties. However, there are reasons to suppose that micromechanics of thin filaments, walls, partitions, as well as the macromechanical behavior of wood may have a lot in common with those in other highly porous materials [148,149,150,151,152,153,154]. Therefore, the general approaches and models developed for the analysis of the latter can be applied to wood as well. Plausibly, such softening depends upon cell wall width, cell morphology, aspect ratio and adhesion, and percentage of the lumens in wood crosscut area.

Some SEs can also be observed at the macro-scale and, to some extent, at meso-scale, though they are less pronounced than that at the nano- or micro-scale. They can be attributed to a growing possibility of large defect (cracks, delaminations, knots, and other wood defects) occurrence in larger objects, and can be described using Weibull statistics [155,156]. However, such a study lies outside the scope of our present review.

## 11. Nanomechanics in Dendrochronology

**Figure 13:**
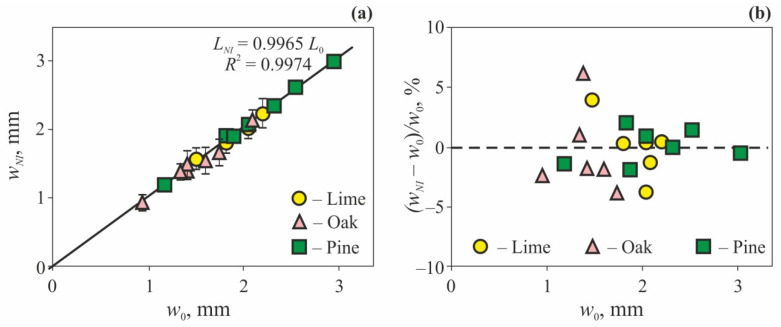
The results of annual growth ring width measurements obtained by nanoindentation *w_NI_* and by the optical method *w*_0_ (**a**), and the discrepancies between these methods (**b**) [117].

Jumps in *E_eff_* and *H_eff_* at the edges of annual growth rings made it possible to measure their width *w_NI_* using scanning NI. Then, the comparison was carried out with the *w*_0_ value determined by an optical method (analysis of image contrast). The image processing method was similar to the one used in the widely used LINTAB equipment. The comparison of the data obtained by these two techniques for measuring tree-ring width is presented in Figure 13; one can see that differences between them do not exceed 2–3% for pine and 4–5% for oak and lime. Mean average deviation for six to seven rings was about 2%. In effect, this means that the scanning indentation method can be used as an alternative to the optical one, or can complement it, providing some additional data on the local mechanical properties.

## 12. Correlation between Thermal Diffusivity and Mechanical Properties of Wood

Kinetic thermophysical characteristics (thermal conductivity λ and diffusivity *a*) and mechanical properties of wood (Young’s modulus, strength, hardness) depend on the same factors, namely on composition, structure, density, porosity, humidity, and specifics of interconnections between microstructural units [5,8,10]. Moreover, in both groups, the same pattern can be observed: higher wood density is accompanied by higher values of the characteristics mentioned above. So, it seems reasonable to suggest that there is a certain correlation between these two groups of properties. Once revealed, such relations could allow switching from laborious and material extensive destructive mechanical tests to non-destructive contactless measurements of λ or *a*. Such approaches could be used to estimate relative mechanical properties, and to sort and grade materials and products made of wood, fiber-reinforced composites, etc.

It should be noted that despite a wealth of information concerning the mechanical and thermal properties of natural and modified wood, wood-based layered materials, as well as composites reinforced with artificial and natural organic fibers, they are measured in separate tests executed on different samples. Exceptions are several papers that employ thermal non-destructive testing to estimate structural damage of wood [157,158], defects [159,160], porosity [161], and anisotropy [162,163] of composites and their possible impact on material mechanical properties. A micromechanical model for the prediction of effective thermal conductivity in two- and three-phase composites is proposed in [164].

The dependence of thermal diffusivity tensor *a_ij_* on Brinelle hardness HB in common pine (*Pinus sylvestris* L.), pedunculate oak (*Quercus robur* L.), and small-leaf lime (*Tilia cordata Mill.*) wood at various humidity levels was studied in [116,165,166].

The thermal diffusivity tensor components *a_ij_* = λ_ij_/*ρC_p_*—where λ_ij_ represents the thermal conductivity tensor components, *ρ* is the material density, and *C_p_* is the specific thermal capacitance—were measured using an original non-destructive thermal imaging technique described in detail in [167,168,169]. The method is based upon local stepped heating at small spots on the sample surface by a focused laser beam while continuously monitoring the surface temperature distribution with a thermal camera. Heat propagation in such a setup is close to spherical symmetry in isotropic materials, while in orthotropic materials, the isothermal surfaces are close to three-axis ellipsoids with the axes fully determined by the main components of *a_ij_* tensor and the time elapsed since heating onset, provided that the distance to the heating center is at least several times higher than the heating beam radius. Therefore, the *a_ij_* values were determined by processing dynamic thermal images obtained on lateral, radial, and transverse crosscuts of wood samples, as described in [167,168,169].

**Figure 14:**
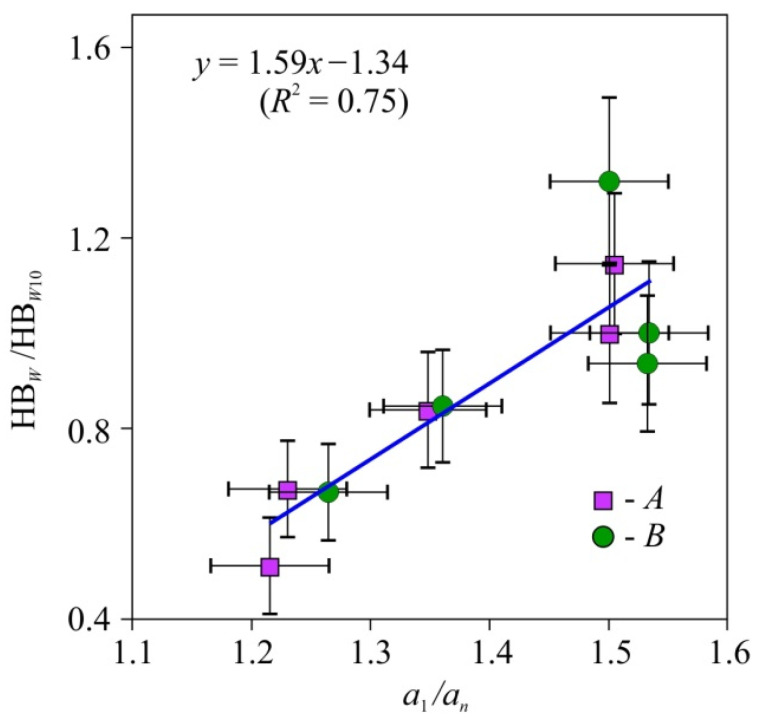
Dependence of the relative macrohardness HB*_W_*/HB*_W_*_10_ of pine wood upon thermal diffusivity anisotropy—*a*_1_/*a_n_*. HB—Brinell hardness (12.7 mm sphere diameter, 1 mm indentation depth), *a*_1_, *a_n_*—thermal diffusivity coefficients along and across the fibers. HB*_W_*/HB*_W_*_10_ is HB*_W_* values on radial (A) and tangential (B) faces, as shown in Figure 2, normalized to HB*_W_* at humidity *W* = 10% (HB*_W_*_10_) [116].

The values of hardness HB*_W_* at current humidity values *W* normalized to the hardness HB*_W10_* at *W* = 10% for the lateral and radial faces were statistically indistinguishable (Figure 14); therefore, they were approximated as a single set by the following linear function (HB*_W_*/HB*_W_*_10_) = 1.59(*a*_l_/*a_n_*) − 1.34 with the coefficient of determination *R*^2^ = 0.75, where *a*_l_ and *a_n_* are longitudinal and lateral components of *a_ij_* tensor, accordingly. The hardness measured on the face perpendicular to the fibers was found to be independent of tensor *a_ii_* components, so that it could be used for method and equipment calibration.

Similar results were obtained for other porous materials. For example, a linear relationship between the coefficient of thermal conductivity λ and compressive strength σ*_b_* in lightweight porous cement composites containing aerogel has been reported in [170]. A decrease in porosity resulted in λ growth from 0.39 to 0.47 W/m∙K, and simultaneous σ*_b_* increase from 7.5 to 15 MPa.

## 13. Discussion

Despite the great variety of plant domain and trees in particular, their structural composition is similar, and this similarity stays across different hierarchical scale levels. They are composed of the same organic molecules that form nanocrystals and nanofibrils, which in turn form microfibers and then cell walls. The tree trunk contains the cells organized in annual growth rings. It is interesting that not only such a structure is more or less universal, but its individual elements in various species have similar morphology and close mechanical properties. Additionally, the higher the scale level in this hierarchy the lower its mechanical characteristics. This decrease is roughly in accordance with the Hall-Petch relation, and this fact has not been noticed before. Reconciling mechanical strength and physiological functions, nature had to find some compromise. Capillaries, large tracheids, and gum canals weaken wood structure inevitably, so that their specifics determine in large part the macromechanical properties of timber products. Relations between wood mechanical properties and the architecture and features of its structure are studied insufficiently yet. In particular, quantitative relations between the polymerization degree of polysaccharides, microfibers and cell walls structure, morphology and shape of the cells, wood microarchitecture, turgor pressure etc., on one hand, and macroscopic mechanical characteristics such as stiffness, hardness, strength, strain at break, and fracture toughness on the other hand, are unknown. Meanwhile, the turgor of a plant cell, for example, averages around 0.44 MPa and can be as high as 2 MPa [35]. It is essential in providing mechanical stability of any plant. It is known that higher cellulose percentage and crystallinity generally provide higher strength and stiffness to microfibers [8,36,40,67,68], as long as other structural components have much lower mechanical characteristics. In particular, hemicellulose stiffness and strength are several tens times lower, and around two orders of magnitude lower for lignin than that of cellulose [36]. Besides, an increase in moisture content leads to much faster deterioration of their mechanical properties than that in cellulose. However, reliable systematic research into cellulose, hemicellulose, lignin, and pectin percentages impacts on wood mechanical properties at different structural levels are lacking. Nevertheless, the role of chemical composition is very significant, especially accounting for the high variance of the above percentages, even in plants of the same species, and more so for plants of differing species [104].

A better understanding of formation regularities of wood hierarchical structure and their relations with mechanical properties could allow for controlling the latter, as well as manufacturing materials with desired properties. In theory, it could be achieved by means of controlled growth of the plant in an artificial environment, modification of wood structure using more efficient techniques, or reconstruction of the material that could provide it with new design and properties. All these approaches are used to some extent already, but without physical substantiation their efficiency is low. The best results have been achieved at nano- and micro-scales by growing nanofibers, and aligning and cross-linking them to form high strength microfibers. A scientifically valid and optimized design of nanocellulose-based materials can fulfill natural potential of this polymer and allow manufacturing ecologically friendly, high-strength, economically efficient materials.

## 14. Conclusions

In spite of the fact that people have been using different kinds of wood and wood derivatives for many thousands of years, the variety of cellulose-containing materials is so vast, and their multilevel architecture is so complicated, that the nature of their properties has not been completely revealed yet. As a result, the potential of these natural materials is not fully used. In order to make accurate predictions about wood characteristics, to be able to control them during growth, or to modify them with various treatments, and to preserve them in service, we need a deep comprehension of which elements of woods’ structure, their condition, orientation, interconnection, and evolution, as well as which defects and flaws in the nano-, micro-, meso-, and macro-structure, influence particular macroscopic properties. First of all, these properties should be understood as the most common and important key elements of wood microstructure, such as nanocrystalline and amorphous phases of cellulose, microfibrils, cell walls, and annual growth rings.

Various modern research methods provide multiscale data on wood mechanical properties at different structural levels, from nano- to macro-scale. The interrelated information from these levels can offer new approaches to the cultivation of wood with predetermined mechanical properties, for example, with high strength and elastic properties, necessary acoustic characteristics, or low creep rate, and would help to create effective physically substantiated methods of wood strengthening. High specific strength of nanocellulose, exceeding that of almost all the other construction materials, except nanocarbons, encourages designing new extra-strong, ecologically friendly materials that would take advantage of this potential.

The results of scanning nano- and micro-mechanical properties of wood across several successive annual growth rings provides the basis for innovative methods and techniques for dendroclimatological and dendrochronological applications that would complement the existing approaches. Since the average cell size in a wood cross-section is about 30–50 μm, and the average ring width is 1–3 mm, there are approximately 50–100 cells in each ring. Technically, nanoindentation provides a means to measure the mechanical characteristics of each individual cell. Therefore, the temporal resolution limit for NI in dendrochronological applications is close to one week, making it a much more precise method with better temporal resolution than the traditional optical ones.

## Figures and Tables

**Table 1:** Structural characteristics of cellulose nanocrystals and individual nanofibrils (CNC and CNF, respectively) [67]. Copyright 2017, Wiley books.

Characteristics	CNCs	CNFs
Length of nanoparticles (nm)	100–500	≥10^3^
Lateral size of nanoparticles (nm)	5–30	10–40
Aspect ratio of nanoparticles	10–50	60–100
Length of crystallites (nm)	70–200	60–150
Lateral size of crystallites (nm)	5–10	3–7
Crystallinity (%)	72–80	50–65
Amorphicity (%)	20–28	35–50
Specific gravity (g cm^−3^)	1.57–1.59	1.54–1.56
Specific volume (cm^3^ g^−1^)	0.63–0.64	0.64–0.65
Porosity (cm^3^ g^−1^)	0.01–0.05	0.1–0.2

**Table 2:** Mechanical characteristics of cellulose nanocrystals and individual nanofibrils (CNC and CNF, respectively) [67]. Copyright 2017, Wiley books.

Characteristics	CNCs	CNFs
Modulus axial (GPa)	140–160	30–40
Modulus transversal (GPa)	15–30	10–15
Tensile strength axial (GPa)	8–10	0.8–1
Tensile strength transversal (GPa)	About 1	About 0.1

**Table 3:** Microfibers Young moduli *E* obtained using different methods for three materials [104].

Sample	Elementary Fibre Tensile Modulus (GPa)	Nanoindentation Modulus (GPa)	AFM Mapping Modulus (GPa)
Eden flax	68.9 ± 24.6	20.4 ± 1.1	21.3 ± 2.2
Bamboo	43.6 ± 0.6	21.3 ± 1.7	21.3 ± 2.9
Tension wood	18–40	14–20	11

## Data Availability

All the data is available within the manuscript.
